# Controversy: Botulinum Toxin in Pregnancy

**DOI:** 10.4103/0974-2077.53091

**Published:** 2009

**Authors:** Munish Paul

**Affiliations:** *Skin Laser Centre, New Delhi, India*

**Keywords:** Botulinum toxin, pregnancy, safety

## Abstract

Botulinum toxin A (BTX-A), a purified protein derived from the bacteria *Clostridium botulinum*, has been widely used in aesthetic dermatology. Though BTX-A was initially used by neurologists extensively for neurological conditions such as blepharospasm, strabismus headaches, dystonia and spasticity, it has become popular among dermatologists and plastic surgeons for its cosmetic indications. Its use in pregnancy has been controversial and this article deals with the issues of use of BTX-A in pregnancy.

## INTRODUCTION

Botulinum toxin – a bacterial extract made of botulinum toxin Type A (BTX-A), a purified protein derived from the bacteria *Clostridium botulinum*, has been widely used in aesthetic dermatology. Though BTX-A was initially used by neurologists extensively for neurological conditions such as blepharospasm, strabismus headaches, dystonia, spasticity, it has become a preferred drug and has been used widely by dermatologists and plastic surgeons for its cosmetic indications.

However, concerns remain about its safety in pregnancy. Considering the widespread use of BTX-A, it is possible that it may be injected unknowingly in pregnant women, especially in the early first trimester when women might not be aware of the pregnancy. Animal studies in mice, rats, rabbits have shown reductions in fetal body weights, delayed ossification, abortions, and/or fetal malformations.[1] There are only a few reports dealing with the issue of safety of BTX-A in human pregnancy.

### Available evidence on the safe use of BTX-A in pregnancy

Newman *et al.*[2] reported the first report of clinical BTX-A treatment during pregnancy. They reported four full-term uncomplicated pregnancies in a patient who received BTX-A treatment during pregnancy for severe cervical dystonia. The patient was injected in each pregnancy in doses ranging from 600-1200 per pregnancy, without any effect on the pregnancy outcome.

Morgan *et al.*[3] surveyed physicians who frequently used BTX-A, for pregnancy outcomes in any of their patients who might, knowingly or unknowingly have been injected with the drug. Twelve physicians reported injecting BTX-A in pregnant women. Sixteen pregnant women had been injected, mostly in the first trimester, and only one patient, who had had history of prior spontaneous abortions, suffered a miscarriage. Another woman had a therapeutic abortion. All other pregnancies went to term and there were no fetal malformations.

De Oliveira Monteiro[4] reported two women who were injected in the early first trimester but had uneventful pregnancies with no untoward effects in the fetus.

## CONCLUSION

Even though these reports indicate that BTX-A may be safe in pregnancy, the reports are few and consist of only a small number of patients. There are no controlled trial data and it is doubtful whether there can ever be such studies conducted to resolve this issue. Administering such a drug for a cosmetic indication is fraught with legal risk. It would therefore be prudent on the part of the treating physician to consider pregnancy as an absolute contraindication for cosmetic BTX-A treatment.
